# Molecular Studies on the Nephroprotective Potential of *Celastrus paniculatus* against Lead-Acetate-Induced Nephrotoxicity in Experimental Rats: Role of the PI3K/AKT Signaling Pathway

**DOI:** 10.3390/molecules26216647

**Published:** 2021-11-02

**Authors:** Karunakaran Balaji, Jagadish Vijayakumar, Ponnusamy Kasirajan Sankaran, Sivanesan Senthilkumar, Rajagopalan Vijayaraghavan, Jayaraman Selvaraj, Maria Francis Yuvaraj

**Affiliations:** 1 Department of Anatomy, Saveetha Institute of Medical & Technical Sciences, Chennai 602105, India; anatomyvijay24@gmail.com (J.V.); sujinalways@gmail.com (M.F.Y.); 2 Department of Anatomy, All India Institute of Medical Sciences, Mangalagiri 522503, India; 3 Department of Research and Development, Saveetha Institute of Medical & Technical Sciences, Chennai 602105, India; senbio@gmail.com (S.S.); jai_vijay@hotmail.com (R.V.); 4 Department of Biochemistry, Saveetha Institute of Medical & Technical Sciences, Saveetha Dental College & Hospital, Chennai 600077, India; selvarajj.sdc@saveetha.com

**Keywords:** *Celastrus paniculatus*, nephroprotective, electron microscopy, lead acetate, nephrotoxicity, P13K/AKT signaling pathway

## Abstract

Chemicals can induce nephrotoxicity, with damage to different segments of the nephron and deterioration of renal function. Nephrotoxicity due to exposure to a toxin such as carbon tetrachloride, sodium oxalate, or heavy metals is the most common cause of kidney injury. The current study aimed to evaluate the protective effects of *Celastrus paniculatus* seed extract against lead-acetate-induced nephrotoxicity by evaluating the histopathology, immunohistochemistry, ultrastructure, and phosphoinositide 3-kinase (PI3K)/protein kinase B (AKT) signaling pathway. Twenty-four rats were divided into four groups (*n* = 6 per group): group 1 contained normal animals and served as the control; group 2 received lead acetate (30 mg/kg body weight (b.w.)/day, oral); group 3 received lead acetate and the standard drug *N*-acetylcysteine (NAC, 200 mg/kg b.w./day, oral); and group 4 received lead acetate and the ethanolic extract of *C. paniculatus* seed (EECP; 800 mg/kg b.w./day, oral). Treatment was given for 28 consecutive days. The data were analyzed using one-way analysis of variance with SIGMA PLOT 13 using SYSTAT software followed by Newman–Keul’s test for comparison between the groups. EECP ameliorated the adverse changes caused by lead acetate. PI3K and AKT messenger RNA (mRNA) levels were diminished in lead-acetate-treated rats. Treatment with EECP inhibited the occurrence of shrunken cells, the atrophy of glomeruli, and degenerative changes in renal tubules caused by lead acetate. Interestingly, the PI3K and AKT mRNA levels were significantly increased in EECP-treated animals. Our results clearly evidence for the first time that *C. paniculatus* seed extract inhibits lead-acetate-induced detrimental changes in kidneys by regulating PI3K/AKT signaling pathways.

## 1. Introduction

Nephrotoxicity due to exposure to a toxin is the most common cause of kidney injury. Any exposure to chemicals such as carbon tetrachloride, sodium oxalate, and heavy metals could lead to nephrotoxicity [1]. Nephrotoxicity is defined as the fast deterioration of normal renal function mainly owing to the toxic effects of chemicals, where damage to different parts of nephrons is seen. About 14–26% of individuals are subjected to drug-induced nephrotoxicity in their lifetime owing to overuse of certain antibodies such as aminoglycosides [2]. According to the Agency for Toxic Substances and Disease Registry (ATSDR), lead can enter the human system by inhalation apart from ingestion, absorption, and injection. About 40% of the exposures are through inhalation from occupation-related exposure, which is absorbed into the intestine and rapidly reaches the circulation, and is deposited in major organs such as the liver, brain, and kidney [3,4]. There is no known safe blood lead concentration level; even concentrations as low as 5 µg/dL may be associated with a developmental defect in children, whereas a level of less than 10 μg/dL (0.48 μmol/L) in an adult is considered to be safe, as per the World Health Organization [5]. Extensive studies and a lengthy history of occupational lead exposure would mislead anyone into thinking that lead toxicity is a long gone entity. Regrettably, this is not the case. Presently, lead is used extensively in the manufacture of acid storage batteries. The demand for lead and its compounds is very high in the automobile sector, mainly thanks to the huge transportation volume after rapid industrialization [6]. Even though there have been abundant experimental studies carried out for many years on the adverse effects of lead acetate (PbA), there is still a knowledge gap in understanding the biological effects of its exposure.

Herbal plants have always been used as the principal constituent of medicine in traditional medicine systems. In India, among alternative medicines, herbs are popular because they help cure illness by synchronizing with the body’s self-defense. A literature review shows that many herbs and extracts have been studied for their nephroprotective property because of the presence of flavonoids and other phenolic compounds [7]. The ethanolic extract of *Celastrus paniculatus* seeds (EECP) is also referred to as the “elixir of life” in traditional medicine thanks to its cognition-enhancing properties [8]. The kidney is the primary target organ of lead toxicity because of its ability to reabsorb and accumulate lead. Because EECP has been reported to possess many biological properties and interesting bioactive compounds, we investigated its protective effect against PbA-induced nephrotoxicity in a rodent model by evaluating the histopathology, immunohistochemistry, ultrastructure, and phosphoinositide 3-kinase (P13K)/protein kinase B (AKT) signaling pathway.

## 2. Results

### 2.1. Effect of EECP on Kidney Histopathology Based on Periodic Acid–Schiff (PAS) Staining 

**Figure 1:**
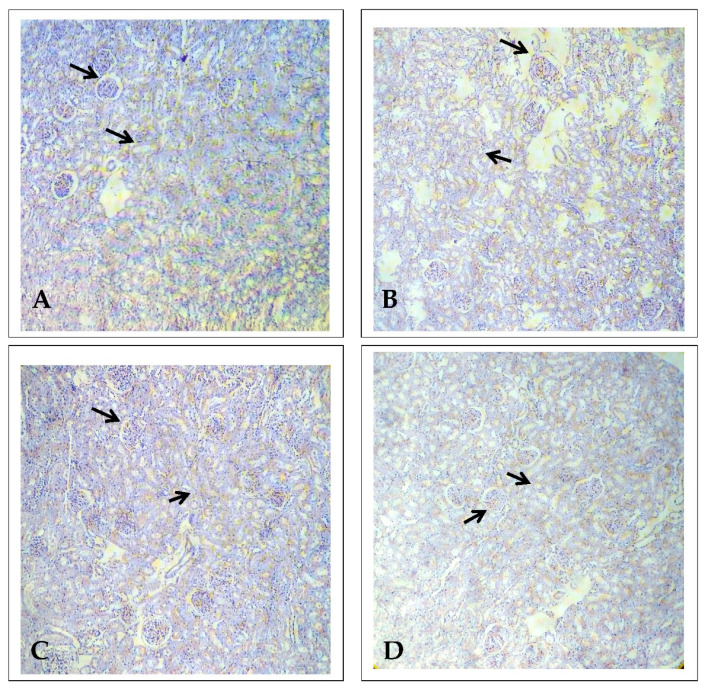
Histopathology of the kidney examined using periodic acid–Schiff (PAS) staining (*n* = 6) (100× magnification). (**A**) Control animals, with well-defined glomeruli with Bowman‘s capsule, and renal tubules. (**B**) Lead acetate (PbA, 30 mg/kg body weight (b.w.)) treated kidney shows shrunken glomeruli with dilated Bowman‘s capsule and degenerated renal tubules. (**C**) *N*-acetylcysteine (NAC; 200 mg/kg b.w.) + PbA (30 mg/kg b.w.) treated kidney shows renal glomeruli and cortical tubules. (**D**) Ethanolic extract of *Celastrus paniculatus* seeds (EECP; 800 mg/kg b.w.) + PbA (30 mg/kg b.w.) treated kidney show normal glomeruli having well-structured Bowman’s capsule, as well as distal and proximal convoluted tubules.

**Figure 2:**
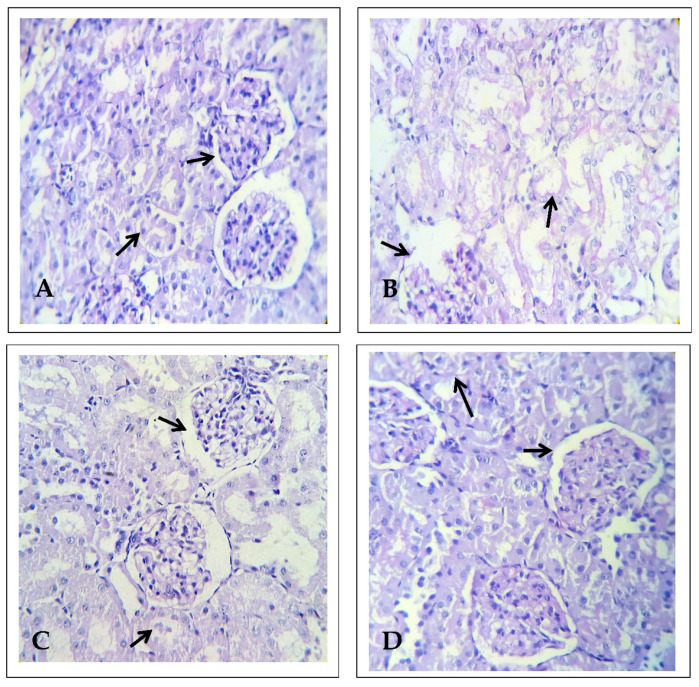
Histopathology of the kidney using periodic acid–Schiff (PAS), (*n* = 6) (400× magnification). (**A**) Control animals, with well-defined glomeruli with Bowman‘s capsule, and renal tubules. (**B**) Lead acetate (PbA, 30 mg/kg body weight (b.w.)) treated kidney shows shrunken glomeruli with dilated Bowman‘s capsule and degenerated renal tubules. (**C**) *N*-acetylcysteine (NAC; 200 mg/kg b.w.) + PbA (30 mg/kg b.w.) treated kidney shows renal glomeruli and cortical tubules. (**D**) Ethanolic extract of Celastrus paniculatus seeds (EECP; 800 mg/kg b.w.) + PbA (30 mg/kg b.w.) treated kidney shows normal glomeruli having a well-structured Bowman’s capsule, as well as distal and proximal convoluted tubules.

Kidney histopathology was examined using PAS staining. The control animals presented a normal architecture showing the features of renal glomeruli and cortical tubules, specifically the convoluted tubule luminal surface lined by cuboidal cells. In this study, PbA (30 mg/kg body weight (b.w.) administrated for 28 consecutive days induced extensive nephrotoxicity (Figure 1 and Figure 2), with significant changes including marked epithelial necrosis and degeneration, moderate to severe interstitial congestion, and inflammatory cell infiltration. In the EECP (800 mg/kg b.w.) and PbA-treated animals, their kidneys showed mild, focal degeneration, and damaged epithelial cells of renal tubules. There were no significant micro-anatomical structural changes noted in the NAC (200 mg/kg b.w.) + PbA-treated animals compared with the kidneys of control animals. Overall, EECP provided substantial nephroprotection against PbA.

### 2.2. Effect of EECP on Kidney Histology Based on Using Masson’s Trichrome Staining

**Figure 3:**
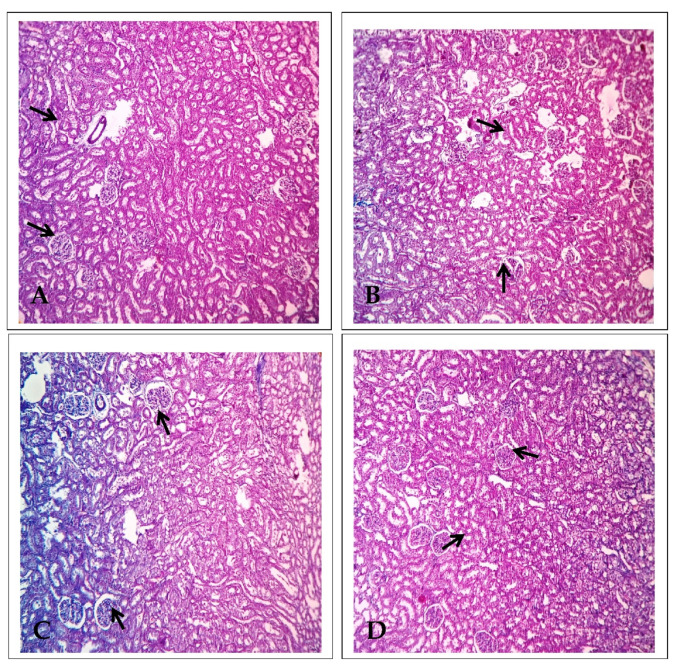
Histopathology of the kidney using Masson’s trichrome stain (*n* = 6) (100× magnification). (**A**) Control kidney shows well-defined glomeruli with a Bowman‘s capsule and renal tubules. (**B**) Lead acetate (PbA; 30 mg/kg body weight (b.w.)) treated kidney shows shrunken glomeruli with a dilated Bowman‘s capsule and degenerated renal tubules. (**C**) *N*-acetylcysteine (NAC; 200 mg/kg b.w.) + PbA (30 mg/kg b.w.) treated kidney shows renal glomeruli and cortical tubules. (**D**) Ethanolic extract of Celastrus paniculatus seeds (EECP; 800 mg/kg b.w.) + PbA (30 mg/kg b.w.) treated kidney shows normal glomeruli having a well-structured Bowman’s capsule as well as distal and proximal convoluted tubules.

**Figure 4:**
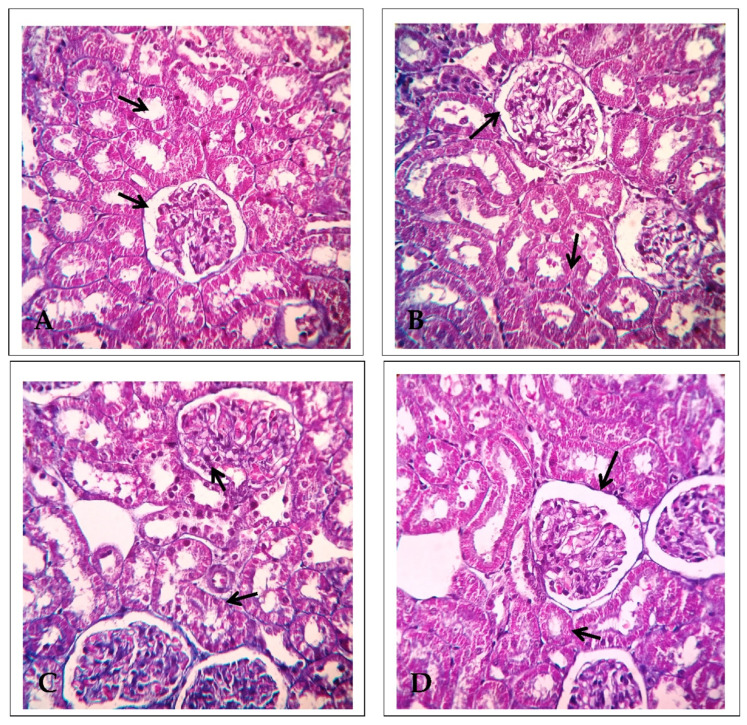
Histopathology of the kidney using Masson’s trichrome stain (*n* = 6) (400× magnification). (**A**) Control kidney shows well-defined glomeruli with a Bowman‘s capsule and renal tubules. (**B**) Lead acetate (PbA; 30 mg/kg body weight (b.w.)) treated kidney shows shrunken glomeruli with a dilated Bowman‘s capsule and degenerated renal tubules. (**C**) *N*-acetylcysteine (NAC; 200 mg/kg b.w.) + PbA (30 mg/kg b.w.) treated kidney shows renal glomeruli and cortical tubules. (**D**) Ethanolic extract of *Celastrus paniculatus* seeds (EECP; 800 mg/kg b.w.) + PbA (30 mg/kg b.w.) treated kidney shows normal glomeruli having a well-structured Bowman’s capsule as well as distal and proximal convoluted tubules.

The kidneys of control and NAC-treated animals showed normal cytoarchitecture of renal structures; that is, properly arranged glomeruli with intact Bowman’s capsule, renal tubules lined with epithelial with an acidophilic cytoplasm, and centrally placed spherical nuclei. The kidneys of PbA-treated animals showed degenerative changes in the proximal and distal convoluted tubule epithelia and congestion of blood vessels. The kidneys of EECP-treated animals showed restoration of the renal structure with mild congestion of blood vessels (Figure 3 and Figure 4). Masson’s trichrome staining precisely outlined the collagen content in the kidneys. There was a significant increase in the total collagen content in the kidneys of PbA-treated animals compared with control animals (Figure 3 and Figure 4). There was also a significant decrease in the total collagen content in the kidneys of EECP + PbA-treated animals compared with PbA-treated animals. Similarly, there was more collagen in the kidneys of animals administrated NAC + PbA compared with those administered PbA (Figure 3 and Figure 4).

### 2.3. Effect of EECP on the Expression of Alpha Glutathione S-Transferase (α-GST) Based on Immunohistochemistry 

**Figure 5:**
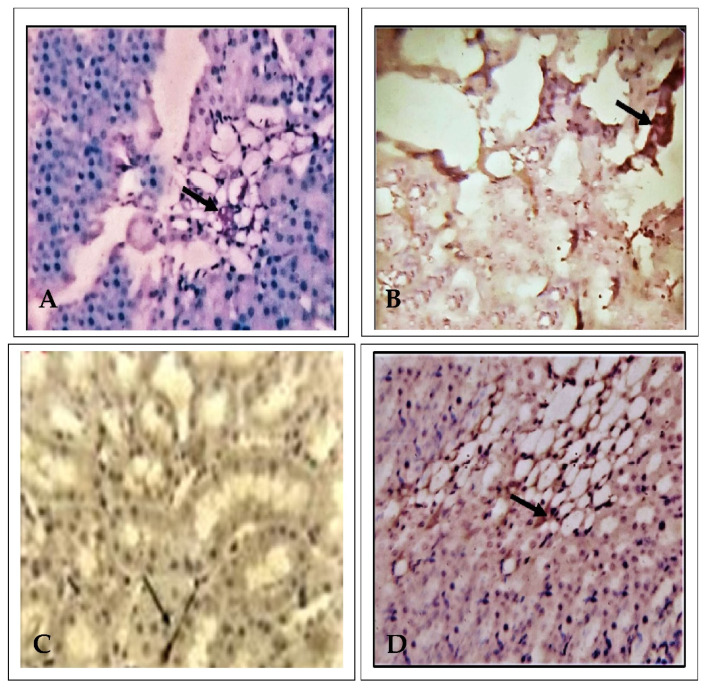
Alpha glutathione S-transferase (α-GST) immunohistochemistry of the kidney (200× magnification). (**A**) Control, (**B**) lead acetate (PbA; 30 mg/kg body weight (b.w.)), (**C**) *N*-acetylcysteine (NAC; 200 mg/kg b.w.) + PbA (30 mg/kg b.w.), and (**D**) ethanolic extract of *Celastrus paniculatus* seeds (EECP; 800 mg/kg b.w.) + PbA (30 mg/kg b.w.). The arrows indicate immunopositive regions.

Figure 5 shows α-GST immunohistochemistry in the kidneys. In PbA-treated animals, there were α-GST-positive cells in the proximal convoluted tubules of the renal cortex and the periphery of the renal medulla. There was very minimal α-GST immune-positivity in the EECP + PbA-treated kidney compared with the PbA-treated kidney. Similarly, the kidneys of NAC + PbA-treated animals showed very few α-GST-positive cells compared with those of PbA-treated animals.

### 2.4. Effect of EECP on the Expression of Delta-Aminolevulinate Dehydratase (ALAD) Using Immunohistochemistry 

**Figure 6:**
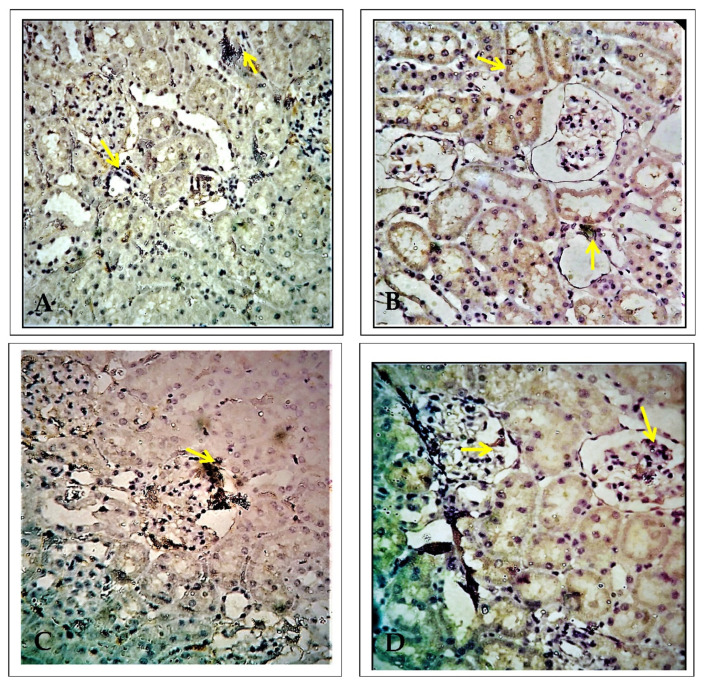
Delta-aminolevulinate dehydratase immunohistochemistry of the kidney (200× magnification). (**A**) Control, (**B**) lead acetate (PbA; 30 mg/kg body weight (b.w.)), (**C**) *N*-acetylcysteine (NAC; 200 mg/kg b.w.) + PbA (30 mg/kg b.w.), and (**D**) ethanolic extract of *Celastrus paniculatus* seeds (EECP; 800 mg/kg b.w.) + PbA (30 mg/kg b.w.). The arrows indicate immunopositive regions.

Figure 6 shows ALAD immunohistochemistry in the kidney of the treated animals. In PbA-administrated animals, ALAD-positive cells were seen in the renal cortex and medulla. There was only mild ALAD positivity in EECP + PbA-treated animals compared with PbA-treated animals; the pattern in the former group was similar to control animals. Similarly, there was no ALAD positivity in NAC + PbA-treated animals compared with PbA-treated animals.

### 2.5. Effect of EECP against PbA-Induced Nephrotoxicity Based on Scanning Electron Microscopy (SEM)

**Figure 7:**
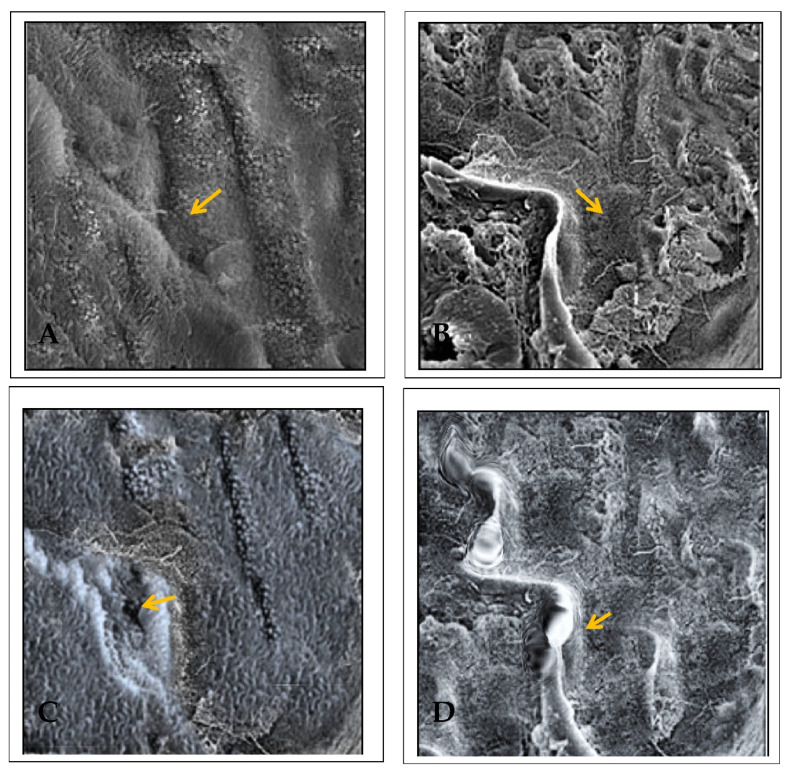
Scanning electron microscopy of the kidney (5000× magnification). (**A**) Control kidney shows the proximal convoluted tubules showed regions of tightly packed microvilli with a proximal tubule brush border. The arrow indicates the lumen and the arrow head indicates the microvilli. (**B**) Lead acetate (PbA; 30 mg/kg body weight (b.w.)) treated kidney shows sparse microvilli and pathological changes (arrow) in the brush border of proximal tubule cells. (**C**) *N*-acetylcysteine (NAC; 200 mg/kg b.w.) + PbA (30 mg/kg b.w.) treated kidney shows large microtubules and tightly packed microvilli. The tubule surface distortion is very much reduced. (**D**) Ethanolic extract of *Celastrus paniculatus* seeds (EECP; 800 mg/kg b.w.) + PbA (30 mg/kg b.w.) shows the tubules was lined with a simple columnar epithelium around a centralized lumen.

The extent of the renal injury in PbA-treated animals was analyzed with SEM. In the control animals, the kidney showed a well-organized cytoarchitecture of the proximal convoluted tubules with regions of tightly packed microvilli with a proximal tubule brush border (Figure 7). PbA administration reduced microvilli and led to pathological changes in the brush border of proximal tubule cells. In the kidney of animals co-administered NAC and PbA, there were large microtubules and tightly packed microvilli with marked renal tubule surface distortion. Finally, the kidney of EECP + PbA-treated animals showed normal renal tubules lined with simple columnar epithelium around a centralized lumen (Figure 7).

### 2.6. Effect of EECP on PI3K and AKT mRNA Levels in the Kidney 

**Figure 8:**
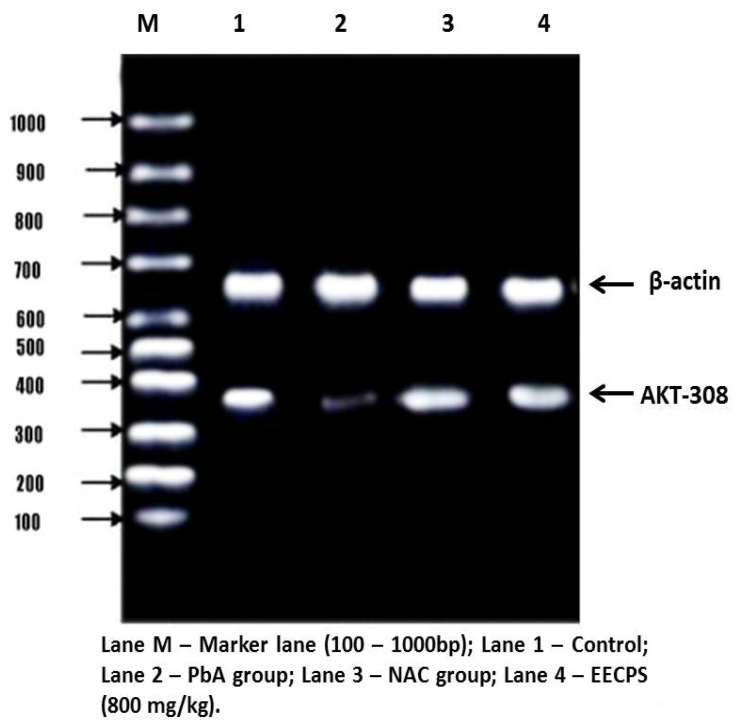
Agarose gel electrophoretic pattern of AKT mRNA expression in the kidney. The lane identities are indicated below the image.

**Figure 9:**
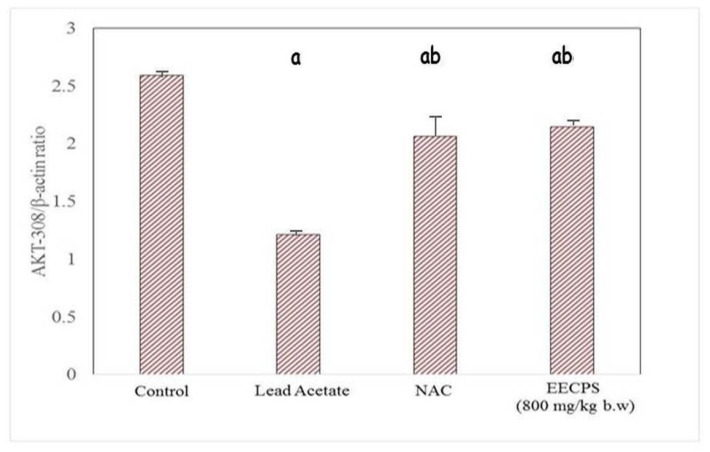
Graphical representation of AKT mRNA expression in the kidney. ^a^ Significantly different from the control group. ^b^ Significantly different from the lead acetate group.

**Figure 10:**
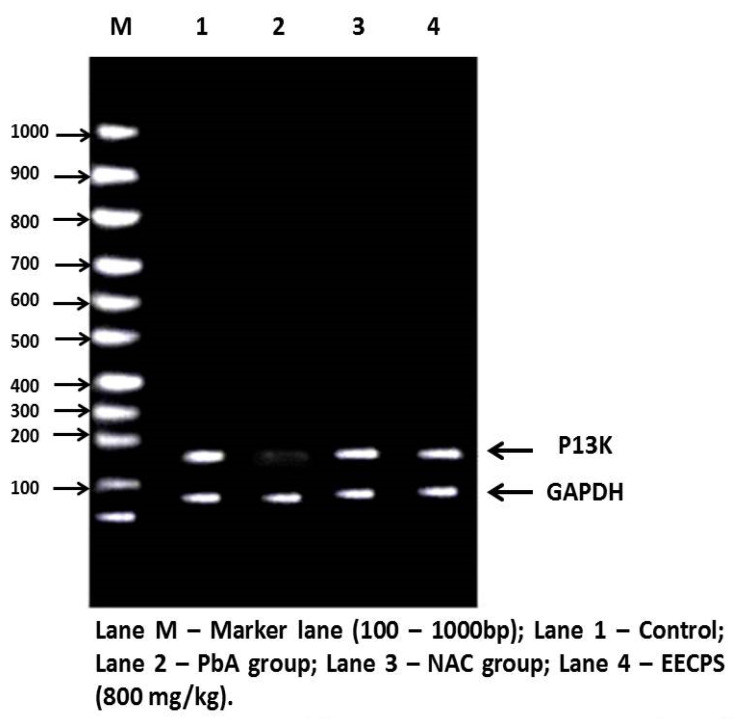
Agarose gel electrophoretic pattern of PI3K mRNA expression in the kidney. The lane identities are indicated below the image.

**Figure 11:**
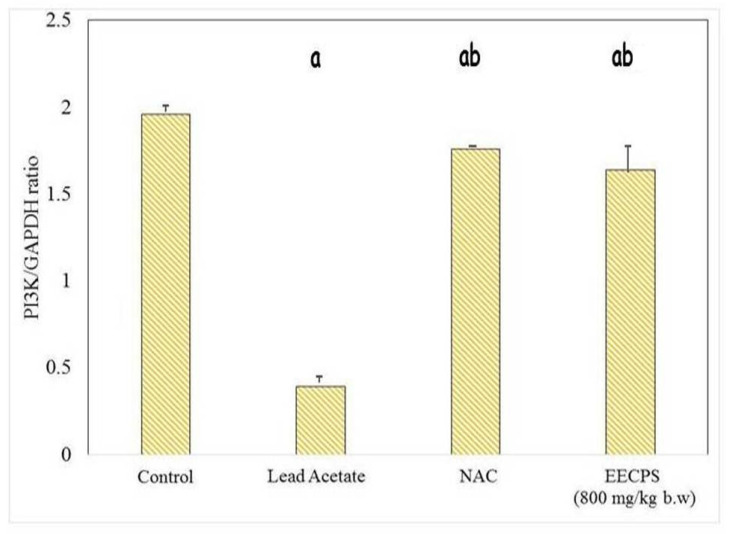
Graphical representation of PI3K mRNA expression in the kidney. ^a^ Significantly different from the control group. ^b^ Significantly different from the lead acetate group.

The PI3K/AKT signaling pathway stimulates cell proliferation, growth, and survival concerning signals from extracellular components. PI3K and AKT are the main proteins in this pathway. PI3K and AKT mRNA levels were significantly reduced in the PbA-treated animals compared with the control animals. In EECP + PbA-treated animals, the levels of both genes were increased significantly compared with the PbA-treated animals. The NAC + PbA-treated animals showed PI3K and AKT gene expression similar to the EECP + PbA-treated animals (Figure 8, Figure 9, Figure 10 and Figure 11).

## 3. Discussion

We found that PbA can cause nephrotoxicity via an adverse effect on the cellular architecture of the kidney. The damage to the renal tissue included epithelial necrosis and degeneration, moderate to severe interstitial congestion, and inflammatory cell infiltration. These histopathological findings are similar to a previous study by Lin et al. [9] after environmental exposure to PbA. Karmakar et al. [10] reported that PbA exposure caused tubular necrosis and inflammatory infiltration. *C. paniculatus* significantly improved the altered renal histopathology to the extent of the control group.

Renal biopsies are becoming a regular and standard procedure in clinical practice. The analysis includes staining with PAS, Masson’s trichrome, and methenamine silver apart from routine hematoxylin and eosin (H&E). The latter is the gold standard procedure in every histopathological laboratory, and the former special stains have been used widely in practice for a long time to better understand the structures damaged in the biopsied tissue [11]. PAS staining is used to study the polysaccharide quantity in tissues, particularly the liver, parathyroid gland, skeletal muscles, and skin. It is also used to evaluate the presentation of the basement membrane and mucus-secreting goblet cells from the epithelia that line several organs. The basic principle for the formation of the magenta color in this stain is the oxidation of carbon–carbon bonds producing aldehydes by periodic acid, which later reacts with the fuchsin-sulfurous acid reagent [12]. The increased release of glucose during ultrafiltration and decreased reabsorption due to damage to the renal tubules results in the accumulation of glucose, which shows more affinity for PAS stain [13,14]. In the kidney interstitial matrix architecture, collagen types I and III are the major constituents. In addition, type IV collagen is present in the basement membrane of the glomeruli of the nephron [15]. Renal damage due to nephrotoxicity leads to an imbalance in the formation and arrangement of collagen fibers, and understanding the extent of this damage could give valuable insight into the severity of the condition [16]. The purpose of using Masson’s trichrome is to differentiate collagen from other structures in the kidney and to check the increase in the collagen content in disease conditions such as nephrotoxicity. By evaluating the kidney in this study with H&E, PAS, and Masson’s trichrome, we found that EECP exerted a protective effect similar to that of NAC. The order of protection was as follows: control > EECP = NAC > PbA.

Apparent renal markers such as KIM-1, NGAL, clusterin, vimentin, uromodulin, nephrin, and netrin are investigated using immunohistochemistry. The early detection of renal damage is vital to protect this organ from progressive and severe complications [17]. α-GST is a sensitive and specific biomarker of proximal convoluted tubular injury of the nephron. Increased α-GST excretion is a sign of acute tubular injury caused by toxic chemicals [18,19]. α-GST is a highly sensitive biomarker for damage related to the permeability of cells and comprises one of the parameters used to assess the response to toxicity. Acute kidney damage leads to the rapid release of high concentrations of α-GST into the bloodstream [20,21]. In our study, there was a marked increase in α-GST immunopositivity in the PbA-treated group compared with the control group. Administration of NAC or EECP concurrently with PbA significantly attenuated this adverse change. EECP counteracted this increase and provided a protective effect against PbA-induced renal damage. Our findings indicate that elevated α-GST could be examined as a specific confirmatory tool for nephrotoxicity.

The biomarkers for lead poisoning are the blood lead concentration, zinc protoporphyrin, and ALAD, a specific and sensitive marker for lead exposure that is also useful to quantify lead exposure [22,23]. A normal value with a broad range and instability are two of the main disadvantages of ALAD [24,25]. Chiu et al. [26] stated that the blood lead concentration was inversely proportional to ALAD activity among lead mining workers with a control group in Taiwan. An ALAD value of 10 g/dL is the threshold value for blood lead level [26]. Feksa et al. [27] presented similar findings in 18 workers exposed to lead because of their occupation. Another study on workers from battery factories showed reduced ALAD with a duration of exposure compared with the control group [28]. There was no significant correlation between ALAD and the blood lead level in urban areas among children in India [29]. Based on the literature, there has been no study determining the ALAD activity using immunohistochemistry in the kidney.

Electron microscopy can provide a comprehensive estimation of the cellular architecture and extracellular framework of the kidney, with detailed information about abnormalities that are not evident based on compound microscopic examination [30]. The electron microscopic study of renal tissue shows the contour, thickness of the glomeruli with foot processes of podocytes, and structural integrity of the basement membrane [31,32]. The occurrence of glomerulonephritis and amyloidosis in patients who received a kidney transplant was studied using an electron microscope with 86% accuracy [33]. Two studies done on 100 cases and 34 cases, respectively, concluded that electron microscopy plays a vital role in the differential diagnosis and prognosis of renal conditions, particularly nephrotic syndrome [34,35]. Another study on 134 cases showed that the combination of immunofluorescence and electron microscopy with light microscopy contributes to the accurate diagnosis of glomerulonephritis [36]. Undoubtedly, the practical use of electron microscopy for renal biopsies is valuable and allows the diagnosis of patient conditions in 48% of cases [37,38]. The primary limitations are that it is time-consuming and costly. Hence, electron microscopy is replaced by immunohistochemistry and used only during negative immunohistochemistry results [39]. The development of segmental glomerulosclerosis during the continuous evaluation of renal tissue in patients with obesity and type 2 diabetes showed defective podocytes with foot process damage associated with protein [40,41,42]. Similarly, the electron microscopic examination revealed injury to foot processes followed by damage to podocytes and resulted in a reduction in the number of podocytes in streptozocin-induced diabetes in Wistar rats [43]. In our study, the scanning electron microscopic analysis showed the loss of structural integrity in the glomeruli, sparse microvilli, and pathological changes observed in the brush border of proximal tubule cells, which was ameliorated by EECP. Indeed, co-administration of EECP restored the microanatomy of renal tissue from PbA-induced toxicity. The flavonoids in *C. paniculatus* seeds likely modulate the adverse effects of PbA to ameliorate the kidney damage. 

The PI3K/AKT pathway regulates cell proliferation, growth, and survival [44,45]. Hepatocytes have high-affinity tyrosine kinase receptors on their cell surface; they phosphorylate P13K to activate it. The phosphorylated PI3K then phosphorylates phosphatidylinositol 4,5-bisphosphate and converts it into active phosphatidylinositol 3,4,5-triphosphate (PIP_3_). Apart from cell growth and survival, AKT also regulates cell glucose metabolism [46]. The phosphorylation of 3-phosphoinositide-dependent kinase 1 with PIP^3^ and PH domain, and similarly the phosphorylation phosphoinositide-dependent kinase 2, activates the AKT protein. The activated AKT downstream of the proteins belong to mammalian target of rapamycin (mTORC) protein syntheses such as Bcl-2, BCL2-associated X, caspase-9 for apoptosis, and glycogen synthase kinase-3 (GSK-3) for glucose metabolism [47]. In that study, PbA downregulated the protein expression of PI3K/AKT. This inhibition led to the elevation of caspase-9 by proapoptotic Bcl-2, which cleaves caspases-3 and caspase-7 to cause apoptosis. In our study, the co-administration of EECP with PbA increased the expression of P13K and AKT mRNA and ameliorated the adverse changes induced by PbA. The order of protection was control > EECP = NAC > PbA. EECP showed a protective effect similar to NAC. The results demonstrate that EECP effectively ceased the PbA-induced renal damage by exhibiting a nephroprotective effect by the above-mentioned mechanisms.

Herbs and their purified components show promising therapeutic potential against various clinical conditions, especially considering that synthetic chemicals may have side effects. Phenolic compounds such as catechin and epicatechin are powerful antioxidants that are present abundantly in grape seeds; they contain anti-aging and anticarcinogenic agents [48]. Many phenolic and alcoholic compounds have been identified in the mulberry fruit, which is widely used in Chinese medicine. Polyphenols, anthocyanins, flavonoids, and more specifically 1-(5-methyl-2-oxy-[1,2,4]oxadiazol-3-yl)-2-phenyl-ethane-1,2-dione 1-oxime, which is found in the mulberry fruit, possess antioxidant potential [49]. The leaves of *Carica papaya*, which is a known antioxidant, were found to have lipophilic and hydrophilic bioactive compounds, and these leaves were also proven to possess a nephroprotective effect against heavy metal such as mercury [50,51]. Similar compounds have been found in *C. paniculatus* seeds; these compounds have been proven to have protective properties in our earlier study [52]. The main limitation of this study is that the bioactive compounds responsible for the nephroprotective effect were not identified, isolated, and purified; instead, the whole plant extract was used. Another limitation is that histopathology was not quantified using histomorphometry. Additional investigation is needed to determine which metabolites of *C. paniculatus* protect the renal tissue from PbA-induced toxicity; is it a single metabolite or the combined activity of two or more components present in *C. paniculatus* seeds?

## 4. Materials and Methods

### 4.1. Chemicals

All chemicals used in the study were of analytical grade, procured from SISCO Research Laboratories Private Limited, D. K. Enterprises, India; Sigma Aldrich, St. Louis, MO, USA; Dako, Carpinteria, CA, USA; or Argutus, Dublin, Ireland. Primers of AKT, PI3K, β-actin, and GAPDH were procured from Eurofins Genomics, Bangalore, India.

### 4.2. Plant Material Preparation and Extraction

*C. paniculatus* seeds were procured from M/s. Herbal Care and Cure Centre, Chennai, and authenticated by a taxonomist of St. Xavier’s College, Tirunelveli. The seeds were subjected to shadow drying and ground into a coarse powder for extraction. Five hundred grams of powder was soaked in 1 L of 90% ethanol. After 72 h, the preparation was filtered and two more separate repeated extractions were done with fresh solvent. The filtrate was combined and evaporated to dryness to obtain a viscous residue. The crude extract was then freeze-dried and stored at −4 °C until further use and named as the ethanolic extract of *C. paniculatus* (EECP). The extract was dissolved in distilled water for oral administration. Earlier reports revealed the extract is not toxic up to a dose of 5000 mg/kg b.w. in rats [53].

### 4.3. Experimental Animals

The study was conducted from September 2019 to February 2020 after proper approval from the institutional animal ethical committee at Saveetha Institute of Medical And Technical Sciences (IAEC Approval No: SU/CLAR/RD/002/2019, dated: 09.08.2019). Female Wistar rats, with a mean weight of 180 ± 20 g, 4–5 months old, were obtained from the Biogen animal facility, Bangalore. Animals were acclimatized for 10 days before the commencement of experiments. They were fed with a standard pellet diet and water ad libitum. The experiment was carried out following the CPCSEA guidelines.

### 4.4. Experimental Design

The animals were divided into four groups (*n* = 6 per group), as described below. The study was carried out for 28 days.

Group 1: Saline (2 mL/kg b.w./day, oral)
Group 2: PbA (30 mg/kg b.w./day, oral)
Group 3: NAC (200 mg/kg b.w./day, oral) + PbA (30 mg/kg b.w./day, oral)
Group 4: EECP (800 mg/kg b.w./day, oral) + PbA (30 mg/kg b.w./day, oral)

### 4.5. Collection of Kdneys

After the study period, on day 29, overnight fasted animals were anesthetized using 1% isoflurane for blood collection and euthanized. A midline incision was made in the undersurface of the abdomen to visualize the organs and harvest the kidneys, which were stored at −80 °C for further analysis. The right kidneys were kept in 10% formalin for histopathological studies, and the left kidneys were used to analyze the antioxidant and oxidative stress markers, as well as gene expression analysis.

### 4.6. Tissue Homogenization

Kidney samples (0.1 g) were homogenized in 1 mL of ice-cold Tris lysis buffer (0.242% 2-amino-2-hydroxymethyl-1,3-propanediol (TRIS) wt/wt in ddH_2_O with 5 mg/mL aprotinin, 5 mg/mL leupeptin, 1 mg/mL pepstatin, and 10 mg/mL phenyl methyl sulfonyl fluoride dissolved in isopropanol) and spun in a Beckman Allegra 6R centrifuge for 20 min at 2100 rpm [54].

### 4.7. Histopathological Studies

After sacrificing animals, the kidneys were harvested and fixed in 10% formalin. The dehydration process was employed by immersing the tissues in a series of ethanolic solutions of increasing concentration to avoid excessive tissue distortion, embedded in the paraffin sections, followed by cutting the sections at a thickness of 3–5 µm with a rotatory microtome. The kidney sections were stained with PAS and Masson’s trichrome. Later, all the slides were observed under 100× and 400× magnification with a compound light microscope. The extent of the morphological changes in the slides was evaluated and photographs were taken.

### 4.8. Expression of α-GST Using Immunohistochemistry

Sections were subjected to antigen retrieval by immersion in citrate buffer (pH 6) at 100 °C for 20 min. Endogenous peroxidase activity was quenched by incubation in 3% hydrogen peroxide in distilled water for 10 min. Endogenous biotin was blocked using a biotin blocking system obtained from Dako. The sections were then incubated with the primary antibodies for 30 min at room temperature. The antibodies were rabbit anti-rat α-GST polyclonal antibody and rabbit anti-dog α-GST polyclonal antibody (Argutus) diluted 1:500. Next, the sections were incubated for 30 min at room temperature with species-specific biotinylated secondary antibodies. The sections were counterstained with hematoxylin, dehydrated, and mounted under glass coverslips. Five 200× microscopic views per slide were selected randomly and photographed using ImageJ software.

### 4.9. Expression of ALAD Using Immunohistochemistry

Immunolocalization of proteins (antigen) in rat kidney tissue was carried out by the indirect peroxidase method. Paraffin was removed in xylene and the sections were dehydrated through a graded alcohol series. After two rinses in phosphate-buffered saline (PBS) for 5 min each, the endogenous peroxidase activity was eliminated by incubation in 3% hydrogen peroxide for 30 min at room temperature. The non-specific binding sites were blocked by incubation with normal goat serum (three drops in 3% bovine serum albumin (BSA) in PBS) for 30 min. After antigen retrieval (100× citrate buffer) for 20 min in a domestic pressure cooker and blocking non-specific binding sites with protein block, the sections were incubated with the primary antibody against ALAD (Thermo Fischer Scientific Company) overnight at 4 °C. For negative controls, the sections were immersed in PBS instead of the specific antibody. The sections were then incubated with primary antibody (1:100) for 60 min at room temperature. After rinsing with PBS, the sections were incubated with biotinylated antiserum (goat anti-rabbit IgG, 1:50 dilution) for 60 min at room temperature [55]. Then, the sections were incubated in the working streptavidin–horseradish peroxidase solution for 60 min at room temperature and washed in three changes of PBS. Finally, the sections were incubated with DAB-hydrogen peroxide for 30 min and washed in water, counterstained, and viewed under a light microscope. For immunohistochemical quantification, two slices were selected from one integration receptor sample. Five 200× microscopic views per slide were selected randomly and photographed using a Nikon ECLIPSE 80i microscope. The area of positive staining was measured in pixels using Image-Pro Software, which detected brown staining in the tissue.

### 4.10. Electron Microscope Study

Kidney sections were fixed with 2.5% glutaraldehyde, dehydrated in ethanol, immersed in liquid nitrogen, and then broken with a razor blade. The tissue pieces were hydrated through graded alcohol washes, washed in sodium cacodylate buffer, and fixed in 1% osmium tetroxide for 1 h. Samples were then washed in buffer, dehydrated, sputter-coated with gold-palladium, and visualized using an FEI-Quanta FEG 200F Microscope [56]. The images were taken at 5000× magnification.

### 4.11. mRNA Expression Analysis by Reverse-Transcription Polymerase Chain Reaction (RT-PCR) 

#### 4.11.1. Isolation of Total RNA

A total of 100 mg of kidney was used for RNA isolation. Total RNA was isolated utilizing Trizol reagent from tissue homogenate (Biobasic Inc.). After RNA isolation, RNA was immediately reverse transcribed into complementary DNA (cDNA) with the Easy Script Plus™ Reverse Transcriptase. It is a novel recombinant reverse transcriptase that displays considerably higher efficiency in the synthesis of the first-strand cDNA from RNA templates.

#### 4.11.2. cDNA Synthesis and PCR Amplification

For RT-PCR, 2 µg of total RNA was employed, and a two-step RT-PCR kit was used. In the first phase, Oligo dT, dNTPs, and reverse transcriptase were used to make cDNA from an RNA template. For 1 h at 37 °C, the components were mixed with DNA primers in a reverse transcriptase buffer. Standard PCR was performed using gene-specific oligonucleotide primers for PI3K, AKT, β-actin, and GAPDH. The procedure started with PCR activation at 95 °C for 5 min; 30 cycles of denaturation at 95 °C for 2 min, annealing at 60 °C for 30 s, and extension at 73 °C for 30 s; and a final extension at 73 °C for 5 min to ensure that the products were fully extended. The housekeeping gene β-actin was co-amplified with gene-specific oligonucleotide primers in the same reaction. The product from each tube (5 µL) was combined with gel loading dye and resolved in a standard 2% agarose gel containing ethidium bromide (0.5 mg/mL) for 2 h under an electrical field of 60 mA and 80 V. A 100 bp molecular weight DNA marker was resolved concurrently. Following electrophoresis, the gel was densitometrically scanned, and the band intensity of each gene of interest was standardized against the band intensity of the housekeeping gene (β-actin) using quantity one software (Bio-Rad, Hercules, CA, USA), before being amplified by PCR. The primer sequences used for the PCR amplification are provided in Table 1 and Table 2.

#### 4.11.3. Agarose Gel Electrophoresis

In a total volume of 25 mL, 1.5% agarose and 1X TAE buffer were prepared and poured onto a gel tray. PCR products were mixed with the loading dye. The mixture was loaded into each well alongside a 1 kb ladder as a reference. The gel was run at 50 V for 90 min and then visualized. Images were digitally captured, and the band intensity was analyzed using Gel Pro Analyzer software, version 4.0. The relative amount of each target gene was normalized to the reference gene, β-actin, and GAPDH [57].

### 4.12. Statistical Analysis

The data obtained from the experiments were analyzed using one-way analysis of variance (ANOVA) with SIGMA PLOT 13 using SYSTAT software followed by the normality test (Shapiro–Wilk test), the equal variance test (Brown–Forsythe), and the multiple comparison test (Newman–Keul’s test) for comparison between the groups. The values are expressed as mean ± standard error of the mean (SEM). *p* < 0.001 was considered statistically significant. The quantitative analysis of immunohistochemistry was performed by counting the number of positive cells in microscope fields using digital ImageJ software.

## 5. Conclusions

In conclusion, PbA induced nephrotoxicity, and EECP attenuated this damage and maintained the general health of the animals. *C. paniculatus* is a potential herbal plant and its seeds are abundant in natural antioxidant compounds and numerous beneficial medicinal properties. The results obtained in this study indicate that *C. paniculatus* seeds possess significant nephron-protecting properties thanks to secondary metabolites present in them. The bioactive compounds of the CP seeds’ extract, such as palmitic acid, ethyl linolenate, and 1-(5-methyl-2-oxy-[1,2,4]oxadiazol-3-yl)-2-phenyl-ethane-1,2-dione 1-oxime, are known to be genuine antioxidants. This indicates that the EECP could have a potential capacity to protect the kidney against PbA-induced toxicity. Identification and isolation of these bioactive compounds could provide useful drugs to overcome lead toxicity.

## Figures and Tables

**Table 1:** AKT-308 primer sequences used for the polymerase chain reaction amplification.

Genes	Forward Primer	Reverse Primer	Product Size (bp)
AKT	5′-ATCCCCTCAACAACTTCTCAGT-3′	R: 5′-CTTCCGTCCACTCTTCTCTTTC-3′	447
β-actin	5′-TGACGGGGTCACCCACACT-3′	5′-CTTAGAAGCATTGCGGTGG-3′	659

**Table 2:** PI3K primer sequences used for the polymerase chain reaction amplification.

Genes	Forward Primer	Reverse Primer	Product Size
PI3K	5′-GTTCACCAATCCTGCCTGTG-3′	5′-CTGCATCACCTTCATCTGGC-3′	248
GAPDH	5′-TGACGGGGTCACCCACACT-3′	5′-CTTAGAAGCATTGCGGTGG-3′	192

## Data Availability

Not applicable.
